# Circulating miRNAs profiles in tourette syndrome: molecular data and clinical implications

**DOI:** 10.1186/s13041-015-0133-y

**Published:** 2015-07-25

**Authors:** Renata Rizzo, Marco Ragusa, Cristina Barbagallo, Mariangela Sammito, Mariangela Gulisano, Paola V Calì, Claudio Pappalardo, Martina Barchitta, Mariagrazia Granata, Angelo G Condorelli, Davide Barbagallo, Marina Scalia, Antonella Agodi, Cinzia Di Pietro, Michele Purrello

**Affiliations:** Section of Child Neurology and Psychiatry, Department of Experimental and Clinical Medicine, University of Catania, Catania, EU Italy; BioMolecular, Genome and Complex Systems BioMedicine Unit (BMGS), Section of Biology and Genetics G Sichel, Department of BioMedicine and BioTechnology, University of Catania, Catania, EU Italy; Department of Medical and Surgical Sciences and Advanced Technologies GF Ingrassia, University of Catania, Catania, EU Italy; Via S Sofia 87, Building C, 2° floor, room 10, 95123 Catania, EU Italy

**Keywords:** Tourette syndrome, Circulating miRNAs, Molecular pathogenesis, Molecular biomarkers, Minimally invasive molecular diagnosis

## Abstract

**Background:**

Tourette Syndrome (TS) is a highly prevalent childhood neuropsychiatric disorder (about 1 %), characterized by multiple motor and one or more vocal tics. The syndrome is commonly associated to comorbid conditions (e.g., Attention Deficit Hyperactivity Disorder and Obsessive Compulsive Disorder), which considerably aggravate clinical symptoms and complicate diagnosis and treatment. To date, TS molecular bases are unknown and its molecular diagnosis is unfeasible.

**Results:**

Due to their master role within cell networks and pathways both in physiology as in pathology, we sought to determine the transcriptome of circulating miRNAs in TS patients: by TaqMan Low Density Arrays, we profiled the expression in serum of 754 miRNAs in six TS patients and three unaffected controls (NCs) (discovery set). These data were validated by single TaqMan assays on serum from 52 TS patients and 15 NCs (validation set). Network and Gene-ontology analysis were performed by using Cytoscape and Babelomics server. We found that miR-429 is significantly underexpressed in TS patients with respect to NCs. Decreased serum levels of miR-429 allowed us to discriminate TS patients from NCs with 95 % of sensitivity and 42 % of specificity. Intriguingly, computational analysis of the network comprising miR-429 targets demonstrates their involvement in differentiation of midbrain and hindbrain and synaptic transmission.

**Conclusions:**

Our data open the way to further molecular characterization of TS and eventual identification of the corresponding genotypes. Circulating miR-429 may be immediately useful as sensitive molecular biomarker to support TS diagnosis, actually based only on DSM-V criteria.

**Electronic supplementary material:**

The online version of this article (doi:10.1186/s13041-015-0133-y) contains supplementary material, which is available to authorized users.

## Background

Gilles de la Tourette Syndrome (TS) is a highly prevalent childhood neurodevelopmental disorder, characterized by both multiple motor and vocal tics: these appear during illness, not necessarily concurrently [1]. Tics are sudden, rapid, recurrent, non rhythmic movements or vocalizations, which wax and wane in frequency; to be diagnostically useful, they must have persisted for more than 1 year since onset (Diagnostic and Statistical Manual of Mental Disorders, DSM-V) [1, 2]. TS prevalence is estimated to be about 1 % [2]. The syndrome is commonly associated to comorbid conditions as Attention Deficit Hyperactivity Disorder (ADHD) and Obsessive Compulsive Disorder (OCD) [2], which have been reported in 60, and 45–60 % of TS patients, respectively [3]: both ADHD and OCD sensibly impair their health-related quality of life [4–6]. The most disabling of them is OCD, which also causes poor self-esteem and low quality of life [7]. It has been stressed that these comorbidities should be considered as negative prognostic factors, given that they considerably worsen prognosis [7–9]. Different TS symptoms are associated with dysfunctions of distinct cortical areas: this has clear implications for the neuroanatomical model of the syndrome [10]. TS has a multifactorial etiology, in which genetic, neurological and environmental factors interact to determine the clinical phenotype [1]. Several genetic mutations have been suggested to be involved in TS (e.g., those at the SLITRK1 *locus* or at *loci* of genes belonging to dopaminergic and serotoninergic pathways) [1, 11]. MicroRNAs (miRNAs) are small non protein-coding RNAs (19–25 nucleotides long), which negatively regulate gene expression at the post-transcriptional level [12, 13]. MiRNAs are master regulators of cellular pathways and networks: accordingly, they perform key roles in different biological processes and diseases [14, 15]. Serum/circulating miRNAs (cmiRNAs) have been detected in all body fluids analyzed to date [16, 17]: this discovery has disclosed the possibility of identifying minimally invasive biomarkers of disease through *liquid* biopsies [18]. In psychiatric diseases, this approach has been already applied to Autism [19], Bipolar Disorder [20], Schizophrenia [21], Depression [22], but not to TS. We hypothesized that altered cmiRNAs serum profiles could be identified also in TS and hint to its biomolecular bases; furthermore and equally important, these molecular data could be immediately applied to strengthen traditional TS diagnosis, actually based on DSM-V criteria. Through a high throughput approach, we identified a cmiRNA differentially expressed (DE) in sera from TS patients with respect to healthy controls. After performing a computational analysis of its target mRNAs, we discovered that these encode proteins that are involved in differentiation of midbrain and hindbrain and synaptic transmission.

## Results

### Demographic data

Fifty eight young caucasian people affected by TS [mean age 12.7 y (SD ±0.9), sex M : F = 50:8] and 18 unaffected controls (NCs) [mean age 12.2 y (SD ± 0.9), sex M : F = 14:4] were recruited into the study. Age distribution was not significantly different within the TS cohort respect to NCs (*p*-value: 0.170). 87.5 % of TS patients and 73.3 % NCs were males: also this difference is not statistically significant (*p*-value 0.178) (Table 1).

**Table 1:** Clinical features

	TS	NCs	*p-*value*
Sex M:F	50:8	14:4	0.178
Mean age	12.7 (±0.9)	12.2 (±2.1)	0.170
Mean age at onset	5.9 (±1.91)		
Ethnic Background: Caucasian	58	18	

TS: Tourette Syndrome

NC: Normal Controls

Standard Deviation is shown between parenthesis

*statistical significance *p* < 0.05

### Neuropsychiatric findings in TS patients

All TS patients presented DCI > 80 % (mean score 87.6 %, SD *±* 7.6). Concerning YGTSS (Yale Global Tic Severity Rating Scale), patients presented a mean total tic score of 29.7 (SD *±* 7.1) and a mean impairment score of 27.9 (SD *±* 8.7). The mean C-YBOCS (Children’s Yale-Brown Obsessive Compulsive Scale) score was 18.3 (SD *±* 8.7) (Table 2). Many patients (39 out 58:67 %) required pharmacological treatment with SSRI, neuroleptics or both. We detected a statistically significant difference in CADS (Conner’s ADHD/DSMV-IV Scale) score between TS patients and NCs (*p*-value 0.000) (Table 2).

**Table 2:** Neuropsychological findings

	TS	NC	*p*-value*
DCI	88.7 % (±7.8)		
YGTSS			
Total Tic Score	29.7 (±7.1)		
Impairment	27.9 (±8.7)		
C-YBOCS	18.3 (±8.7)		
CONNERS	18.8 (±2.7)	7.5 (±2.4)	0.000
CDI	8.6 (±5.4)	7.3 (±2.6)	0.370
MASC	44.8 (±13.7)	41.8 (±17.2)	0.478
CBCL			
Tot	32.7 (±7.4)	22.1 (±6.2)	0.000
Int	9.9 (±2.9)	8.1 (±2.2)	0.028
Ext	10.9 (±2.6)	8.8 (±2.7)	0.002

DCI: Diagnostic Confidence Index

YGTSS: Yale Global Tic Severity Scale

C-YBOCS: Children-Yale Brown Obsessive Compulsive Scale

CDI: Child Depression Inventory

MASC: Multidimensional Anxiety Scale for Children

CBCL: Child Behavior Check-List; Tot: Total; Int: Internalizing; Ext: Externalizing

Standard Deviation is shown between parenthesis

*statistical significance *p* < 0.05

### MiRNAs profile in TS patients

Through TaqMan Low Density Array (TLDA) analysis, we determined the total expression profiles of 754 miRNAs in sera from six TS patients and three unaffected controls. Real time experiments were separately performed for fluidic card A and B: each card contained 384 TaqMan assays. Cycle thresholds (Cts) obtained for each sample for each miRNA were used to calculated DCt, which were computed by the SAM method to identify differentially expressed (DE) miRNAs (Additional file 1). We found that only miR-429 was differentially expressed: it was significantly downregulated in serum of TS patients with respect to NCs by using both GMN method and miR-320 as endogenous control (FDR = 0.024).

### Validation by single TaqMan assays

**Fig. 1:**
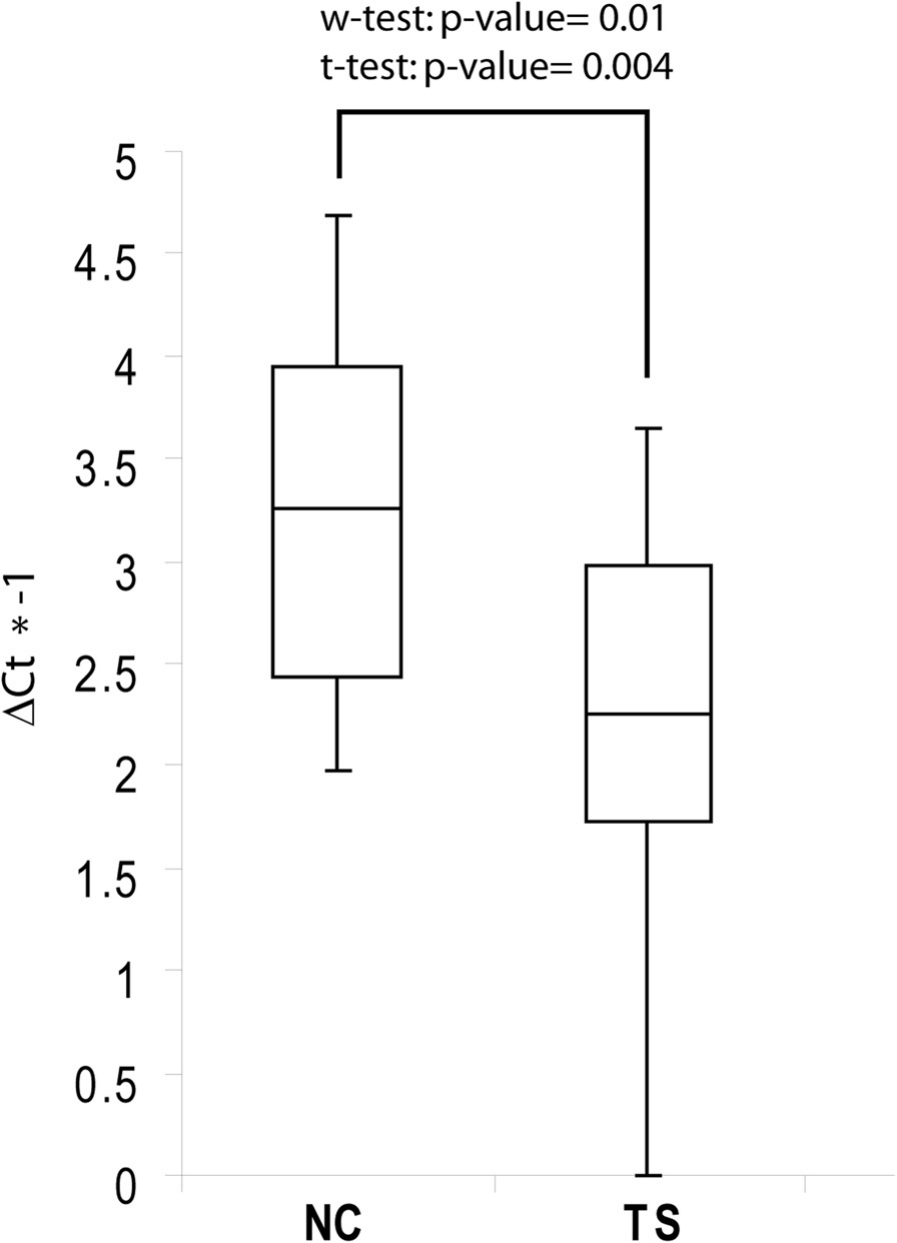
Single TaqMan assays for miR-429. Box plots describing the expression of miR-429 in TS patients and NCs. y-axis represents the-ΔCt of miRNA. *P*-values for Wilcoxon rank sum test and *t*-test are reported above the boxes

**Fig. 2:**
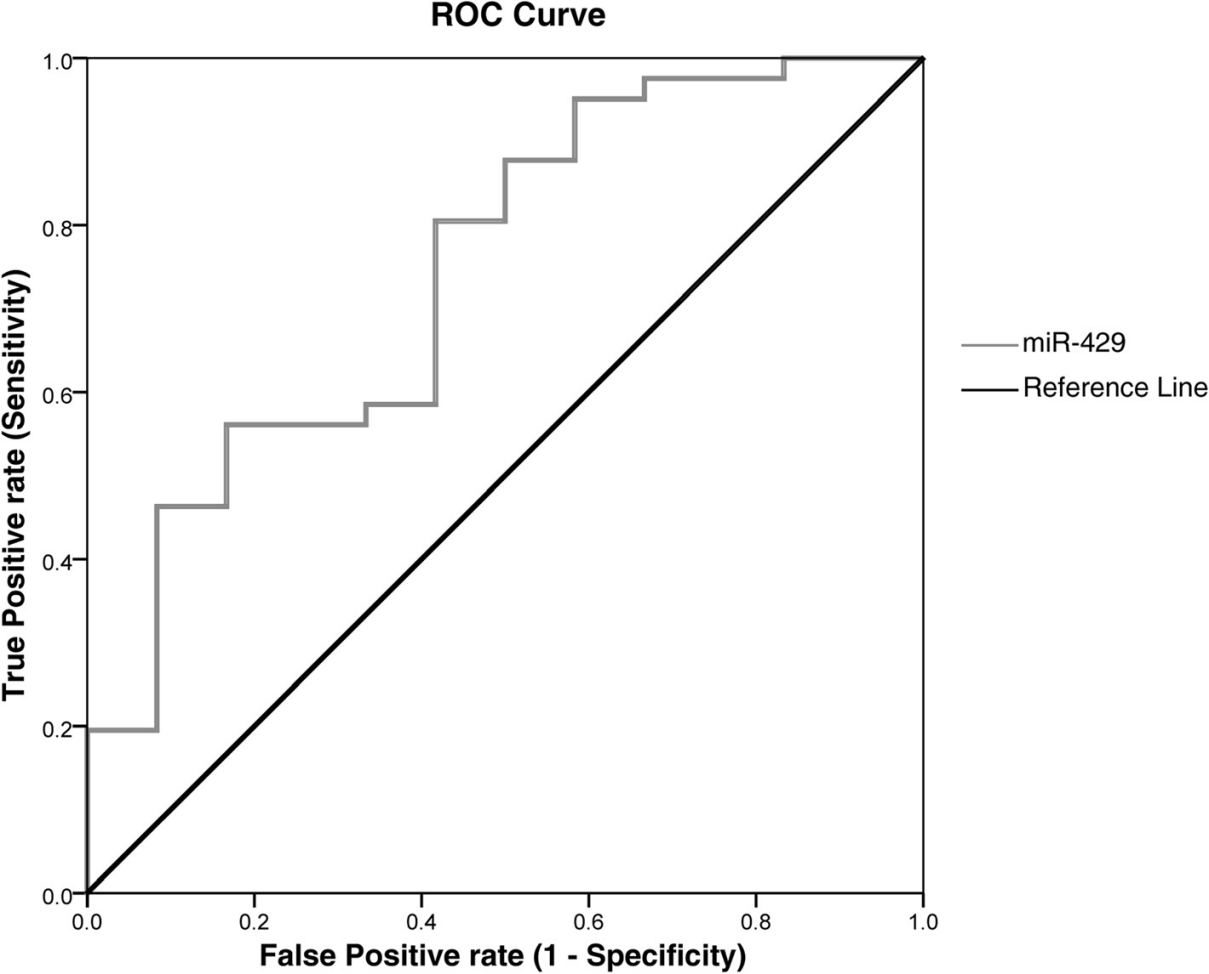
Receiver Operator Characteristic (ROC) Curve for miR-429 expression in TS patients. ROC curve of miR-429 DCts for TS detection. Gray curve represents DCts calculated by using miR-320 as endogenous control

Expression of miR-429 was subjected to validation through single TaqMan assays in serum of 52 TS patients and 15 NCs. By applying both parametric (*t*-test) and non parametric (Wilcoxon test) tests, we confirmed the statistically significant downregulation of miR-429 in TS patients with respect to NCs (Wilcoxon test *p*-value = 0.01; *t*-test *p*-value = 0.004) (Fig. 1). By computing a ROC curve, we found that the decrease of miR-429 serum levels was able to discriminate TS patients from NCs. Specifically, we obtained an AUC of 0.75 (95 % CI, 0.584‑0.907; *p* = 0.01), with 95 % of sensitivity and 42 % of specificity (DCt cut-off value: 10.32) (Fig. 2).

### Correlation between miR-429 expression and neuropsychiatric parameters

**Fig. 3:**
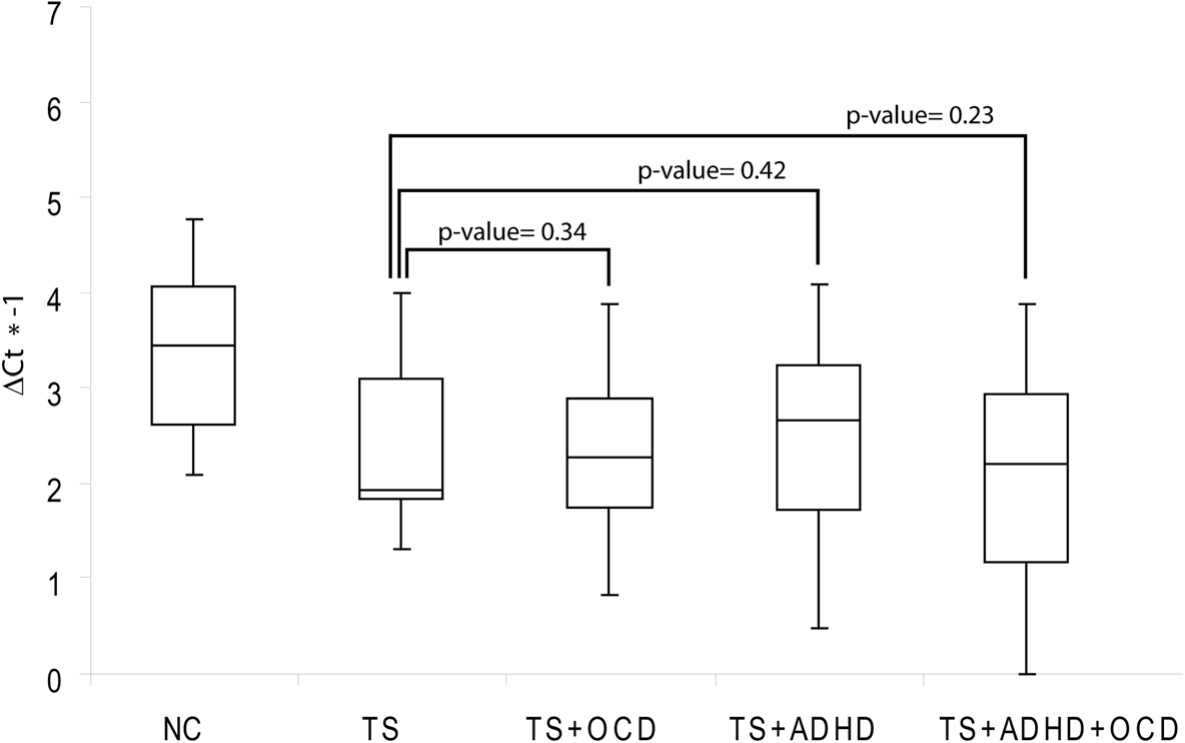
Correlation between miR-429 expression and clinical parameters. Box plots describing the expression of miR-429 in TS patients separated in comorbidity groups and NCs. y-axis represents the –ΔCt of miRNA. *P*-values for *t*-test are reported above the boxes

To evaluate whether expression changes of miR-429 were associated with the clinical and pathologic characteristics observed in patients, its expression levels were compared to different parameters including C-YBOCS, YGTSS, Conner’s ADHD. However, we found only a weak positive correlation between Conner’s ADHD and miR-429 expression (Pearson = 0.28; *p*value = 0.03), while there were no significant associations among miRNAs expression and C-YBOCS and YGTSS scores. Next, we assigned the expression levels of miR-429 to comorbidity groups: (TS + OCD: 20 patients), (TS + ADHD: 13 patients), (TS + ADHD + OCD: 16 patients). We compared the expression of miR-429 among different comorbidity groups and patients with no comorbidity (Fig. 3). We found no statistically significant difference of expression among these groups, although patients with TS+ADHD+OCD showed a downregulation of miR-429 slightly more pronounced than other groups.

### MiRNA targets identification and network analysis

**Fig. 4:**
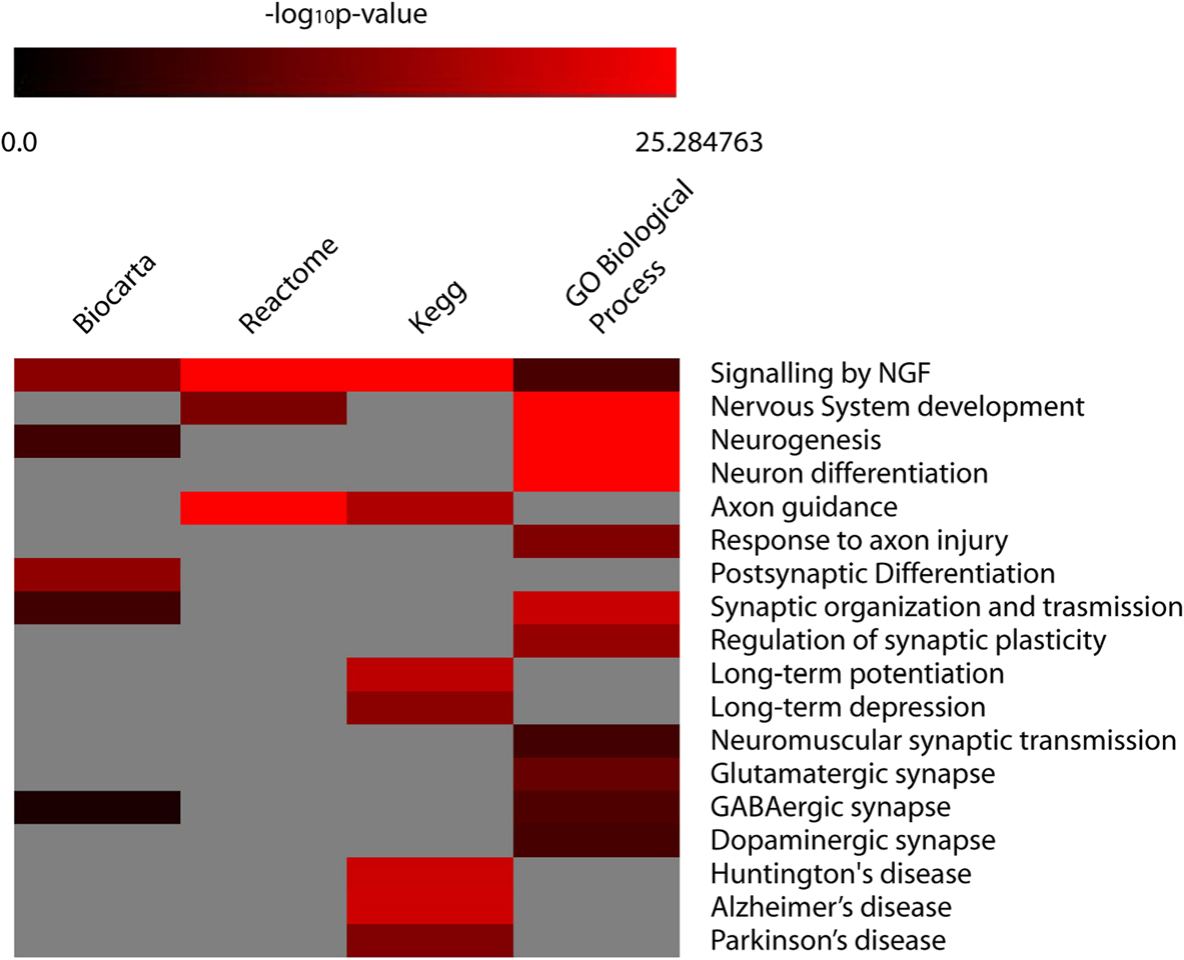
Biological Processes controlled by miR-429 network. Overrepresented biological functions from a molecular network built on validated miR-429 targets, retrieved by different annotation databases (Biocarta, Reactome, KEGG, GO). Data are shown as – log10 of *p*-values for each biological process

To pinpoint the biological functions of miR-429 and rationalize its etiological involvement in TS, we computationally retrieved its validated targets (26 targets based on TarBase); we then built a molecular network comprising miR-429 validated targets and their first neighbours. The generated network, consisting of 1472 nodes and 11,532 edges, was topologically centred on BCL2, CREBBP, EP300, HNF4A, MYC (network nodes with highest degree and betweenness). By using FatiGO enrichment analysis tool, we compared the statistically overrepresented biological functions of all nodes within the network against the entire genome; the following databases were analyzed: Biocarta, Reactome, KEGG, GO. We found that miR-429 directly or indirectly interacts with proteins involved in processes related to physiological and pathological functions of the nervous system (e.g., development and differentiation of neurons, synaptic and post-synaptic transmission and plasticity, neurodegenerative diseases), (Hypergeometric test; Benjamini & Hochberg FDR Correction; *p* ≤ 0.01) (Fig. 4).

## Discussion

Within cells, miRNAs perform a critical biomolecular role as master regulators of networks and pathways. Numerous studies have demonstrated that the genes encoding them are causally implicated in neurodegenerative diseases (e.g., Parkinson’s Disease [23], Alzheimer’s Disease [24], Huntington’s Chorea [25], Frontotemporal Dementia [26]). They also are causally involved in neuropsychiatric disorders (e.g., Schizophrenia [27], Bipolar Disorder [28], Autism [29]). Circulating miRNAs have been already reported as potential markers of these diseases [18–21]. Tourette syndrome was the first neuropsychiatric disease etiologically linked to miRNAs, when TS patients were reported to have a mutation in the binding site of miR-189 at the 3’ UTR of the SLITRK1 gene [30]. This finding was not followed by further characterization of miRNAs role in TS; furthermore, no data on cmiRNAs related to TS have been published to date. Notwithstanding the uncertainty on their tissues of origin, the discovery of cmiRNAs has opened new exciting research perspectives for the identification of the biomolecular bases of pathologic phenotypes [16, 31, 32]; it also promises important translational applications to clinical diagnosis and therapy [33, 34]. In fact, cmiRNAs are endowed with many characteristics that confer them the properties of good biomarkers. They are easily detectable by commonly applied analytical methods as qRT-PCR [34]. They also are much more stable than mRNAs in body fluids, possibly due also to their inclusion in nucleoprotein complexes [35] or encapsulation in microvescicles and exosomes [36, 37]: accordingly, cmiRNAs are resistant to harsh condition, as multiple cycles of freezing - thawing, boiling, very low or high pH, extended storage. Profiling data on cmiRNAs in patients with neuropsychiatric disorders have been already reported for bipolar disorder [20], schizophrenia [21] and depression [22]. For these pathologies, clinical diagnosis is currently based on DSM-V criteria (Diagnostic and Statistical Manual of Mental Disorders, fifth edition): profiling of cmiRNAs, after a *liquid biopsy*, offers a new potentially very important molecular diagnostic tool. Our study is the first report on profiling of miRNAs transcriptome in serum from TS patients. Our experiments allowed us to identify the statistically significant downregulation of cmiR-429 in serum from TS patients: this allowed us to discriminate TS patients from unaffected controls. In fact, ROC curve analysis suggests that this miRNA could be considered a good marker to diagnose TS, as its sensitivity is 95 % and its specificity is 42 %. Finally, our data show a statistically significant positive association between miR-429 expression and Conner’s ADHD, suggesting a molecular relationship between biological processes regulating attentional control and inhibitory control (e.g., dopamine and norepinephrine pathways) and circulating miRNA expression. MiR-429 is a member of miR-200 family (i.e., miR-141, -200a, -200b, -200c, miR-429), which has been shown to inhibit the epithelial–mesenchymal transition by downregulating ZEB1 and ZEB2 [38, 39]. Interestingly, the genes encoding miR-200a, miR-200b and miR-429 itself are located at 1p36.33, a genomic region with copy number variants that has been recently associated to Autism [40, 41]. The potential involvement of miR-429 in Autism is intriguing, as it has been suggested that TS, ADHD, ASD (Autism Spectrum Disorders), and OCD share a common genetic background [42, 43]. By directly targeting the pluripotency factor Sox2 and the cell-cycle regulator E2F3 in neural stem/progenitor cells, members of miR-200 family promote cell-cycle exit and neuronal differentiation of ventral midbrain/hindbrain neural progenitors, including midbrain dopaminergic neurons [44]. Further, data on mice treated with cocaine suggest that the expression of miR-429 could be involved in dendritic morphology and synaptic plasticity [45]. Altogether, these data strongly suggest that miR-429 may be involved in molecular circuits, which control several aspects of midbrain and hindbrain differentiation and synaptic transmission: this is nicely highlighted by our computational analysis of the molecular network centered on miR-429 targets (Fig. 4). The potential importance of these results is stressed by the results of several studies, which have reported alterations in TS of molecular pathways controlling regional boundary formation during brain development, including their essential role in midbrain-hindbrain boundary formation [46–48], and proposed that dopaminergic and serotonergic genes are involved in TS etiopathogenesis [49].

## Conclusions

Based on our data, we propose that miR-429 is a promising biomarker for minimally invasive molecular diagnosis of TS: accordingly, it should be added to classical diagnostic clinical methodologies. Interestingly, our network analysis shows that miR-429 targets are involved in critical aspects of neurodifferentiation and synaptic transmission: these findings open the way to further molecular characterisation of TS and eventual identification of TS genotypes.

## Methods

### Ethics Statement

This study was conducted according to the Declaration of Helsinki and was approved by the Institutional Review Board of the University of Catania; a written informed consent was obtained from each study participant.

### Patients recruitment

Peripheral blood samples were collected from 58 young Caucasian people affected by TS [age range 6–17 y; mean age 12.7 y (SD ±0.9), sex M:F = 50:8] and compared with 18 NCs [age range 10–15 y; mean age 12.2 y (SD ± 0.9), sex M:F = 14:4]. TS patients presented an age of onset of 5.9 years (SD ± 1.91): they were recruited and clinically studied at the Neuropsychiatric Unit, Section of Child Neurology and Psychiatry, Department of Clinical and Experimental Medicine, University of Catania, Catania, Italy, EU. NCs were volunteers recruited from local schools. Demographics are reported in Table 1.

### Neuropsychological procedures

Upon collecting basic demographic data, diagnosis of TS and associated clinical conditions was made in accordance to DSM-V criteria by child neurologists with extensive specific experience (RR and her Collaborators). All TS patients were initially assessed by using a semi-structured interview (i.e., the National Hospital Interview Schedule for Gilles de la Tourette Syndrome) [50] and DCI [51]. Patients with TS and NCs completed the WISC-III [52], YQOL-R [53], MASC [54], CDI [55], CADS [56], and the Child Behavior Checklist (CBCL) [57]. MASC and CDI were used to assess anxiety and depression, CADS provided an indication of symptoms related to ADHD, and CBCL was used to assess a range of emotional and behavioral difficulties. TS patients underwent two additional clinician-rated interviews: the YGTSS [58], to measure the severity of tics, and the Y-BOCS [59] to assess symptoms related to OCD. The YGTSS is an 11-item clinician-rated interview of motor and phonic tic severity. The clinician initially notes the presence of motor and phonic tics based on child and parent report over the past week, as well as behavioral observations. Following this, the clinician rates the severity of motor and phonic tics on five separate dimensions each: number, frequency, intensity, complexity and interference. These scales score rating from 0 to 50. The YGTSS also includes a separate impairment rating scale also rated from 0 to 50. Higher scores indicate the presence of severe symptoms and impairment. The CY-BOCS is the most used and reproducible instrument to assess the severity of obsessive-compulsive symptoms in children. The clinician initially notes the presence of obsessions and compulsions based on child and parent report over the past week, as well as behavioral observations. Following this, the clinician rates the severity of obsessions and compulsions on separate dimensions each: number, frequency, intensity, resistance and interference. Three index scores are obtained: Obsession score, Compulsion score and Total score. The total score evaluates the impairment that obsessive - compulsive symptoms caused to the patient. Based on the total score, the results can be interpreted as follows: 0–7 sub-clinical; 8–15 mild; 16–23 moderate; 24–31 severe; 32–40 extreme. Patients scoring in the mild range or higher are likely experiencing a significant negative impact on their quality of life.

### Entry criteria

We included in this study TS patients who presented normal IQ (tested with Wechsler Scale). Initial diagnosis was confirmed after one year: patients who were unable to be fully assessed at the initial evaluation and/or at follow-up were excluded. Moreover, we excluded all patients who showed evidence of severe neurological or physical impairment. Our cohort was comprised of TS patients who presented DCI > 80 %. NCs were defined as individuals who had neither chronic diseases nor psychiatric disorders; they were matched for age, sex and ethnic group.

### Sample processing

Peripheral blood samples (3 ml) from patients and controls were drawn in the morning, using a butterfly device inserted into collection tubes equipped with Clot activator and gel for serum separation (BD Biosciences). Collection tubes were treated according to current procedures for clinical samples [60]. To separate serum from cellular components, tubes were rotated end-over-end at 20 °C for 30’; they were then centrifuged at 4000 rpm in a Beckman J2-21, at 4 °C for 15’, to spin down blood cells. Supernatant was isolated and centrifuged again at 5000 rpm, at 4 °C for 10’, to remove circulating cells or debris [61]. Serum samples were aliquoted into 1.5 mL, RNase-free, Eppendorf tubes and stored at −80 °C until analysis. Sera were analyzed with a Multiscan Ascent microplate reader spectrometer (Thermo Fisher Scientific) at λ = nm 414, setting an absorbance value < 0.2 as cut-off [61], to distinguish haemolyzed from non-haemolyzed sera.

### RNA extraction

RNA was extracted from 400 μl serum samples by using a *Qiagen miRNeasy Mini Kit (Qiagen, GmbH, Hilden, Germany)*, according to Qiagen Supplementary Protocol for purification of small RNAs from serum and plasma. RNA was eluted in a 40 μl total volume of elution buffer with two consecutive steps of elution (30 ul followed by other 10 μl) in the same collection tube. RNAs were quantified by spectrophotometry.

### miRNAs profiling

To profile the transcriptome of 754 different human miRNAs, 4.5 μl of RNA (corresponding to 30 ng of RNA) were retrotranscribed and preamplified according to manufacturer’s instructions. Preamplified products were loaded on TLDA, TaqMan Human MicroRNA Array v3.0 A and B (Applied Biosystems, Foster City, CA). PCR reactions on TLDA were performed on a 7900HT Fast Real Time PCR System (Applied Biosystems). To obtain an accurate miRNA profiling, we used the global median normalization (GMN) method. Similar to microarray analysis, Ct values from each sample were normalized to the median Ct of the array [62, 63]. By computing the Pearson correlation among the Ct medians and means of each array and Ct of each miRNA, we identified a miRNA that showed an expression profile closer to the median and mean of TLDAs: miR-320. Expression fold changes were calculated by the 2 − ΔΔCT method [62, 63]. Differentially Expressed (DE) miRNAs were identified by Significance of Microarrays Analysis (SAM) (http://www.tm4.org), applying an unpaired test among ΔCt and using a p-value based on 100 permutations; imputation engine: K-nearest neighbours (10 neighbours); false discovery rate (FDR) < 0.15.

### Validation with single Taqman assays

Purified RNAs from sera of 52 TS patients and 15 NCs (validation set) were used for miRNA-specific reverse transcription to obtain miRNA-specific cDNAs. RT-PCR analysis was performed by using TaqMan MicroRNA Assays (Applied Biosystems) specific for DE miRNAs and for controls.

### Statistical analyses

All statistical analyses were performed using the SPSS software (Version 22.0, SPSS, Chicago, IL). Parametric independent Student *t*-test and non parametric Wilcoxon test were used to compare miRNAs serum levels between the TS patients and NCs. DCts for miR-429 respect to endogenous control miR-320 were used to generate a Receiver Operating Characteristic (ROC) curve. Area Under the Curve (AUC) and 95 % confidence intervals (95 % CIs) were calculated to assess the accuracy of each parameter (sensitivity and specificity) and to find an appropriate cut-off point. Statistical significance was established at a *p*-value ≤ 0.05.

### Computational Analysis

Validated targets of miR-429 were retrieved by miRTarBase (http://mirtarbase.mbc.nctu.edu.tw/). The corresponding biological network was built by retrieving interactome data through MiMi Plugin of cytoscape (http://mimiplugin.ncibi.org/). The tool FatiGO (http://babelomics3.bioinfo.cipf.es) was applied to determined the statistical overrepresentation for Gene Ontology (GO), KEGG and BioCarta terms of miRNAs targets network.

## Additional file

Additional file 1:
**Delta Ct obtained by global median normalization method for TLDA Panel A and B.** (XLSX 72 kb)

## Abbreviations

ADHD: Attention deficit hyperactivity disorder
ASD: Autism spectrum disorders
AUC: Area under the curve
CADS: Conner’s ADHD/DSMV-IV scale
CBCL: Child behavior checklist
CDI: Child depression inventory
cmiRNAs: circulating miRNAs
DCI: Diagnostic confidence index
DE: Differentially expressed
FDR: False discovery rate
MASC: Multidimensional anxiety scale for children
NCs: Unaffected controls
OCD: Obsessive compulsive disorder
SD: Standard deviation
TLDA: TaqMan low density array
TS: Tourette syndrome
WISC-III: Wechsler intelligence scale for children III
Y-BOCS: Children’s yale- brown obsessive compulsive scale
YGTSS: Yale global tic severity rating scale
YQOL-R: Youth quality of life instrument-research version
